# Diabetic Ketoacidosis Presenting as Abdominal Pain in a Two-Year-Old Female: A Case Report

**DOI:** 10.7759/cureus.61730

**Published:** 2024-06-05

**Authors:** Samantha Boever, Angela Johansson

**Affiliations:** 1 Pediatrics, A.T. Still University, Mesa, USA; 2 Pediatrics, Adelante Healthcare, Surprise, USA

**Keywords:** type 1 diabetes mellitus (t1d), high anion gap metabolic acidosis, pseudo-hyponatremia, pediatric diabetes, diabetic keto acidosis

## Abstract

Diabetic ketoacidosis (DKA) is a potentially life-threatening complication of diabetes and can sometimes be the first indication of undiagnosed type 1 diabetes mellitus (T1DM). Our case presents a unique scenario in which a two-year-old female presented to her pediatrician with persistent abdominal pain, along with fatigue and tachypnea. On physical examination, she was mildly distressed, tachypneic, and utilized accessory muscles during respiration. Subsequent urinalysis indicated elevated glucose levels of 500 milligrams/deciliter (mg/dL). She was promptly referred to the emergency department to be treated for DKA. Upon arrival, the patient's glucose level was elevated at 533 mg/dL, with an anion gap of 25. She was stabilized and admitted to the pediatric intensive care unit (PICU) with a new diagnosis of T1DM with ketoacidosis. Given the emergent nature of DKA and the need for immediate treatment, physicians should consider DKA as a potential diagnosis for any pediatric patient presenting with abdominal pain.

## Introduction

Diabetic ketoacidosis (DKA), a life-threatening complication that can arise in the context of new-onset type one diabetes mellitus (T1DM), stands as the primary cause of hospitalization, morbidity, and mortality among children below the age of 15 [1]. DKA may also occur in T1DM patients who are acutely ill, who neglect to administer short-acting insulin before meals (improper bolus dosing), or who experience other dose-related issues such as excessive or insufficient insulin administration at inappropriate times. Individuals below the age of two and those with constrained financial resources are more vulnerable to developing DKA during the initial diagnosis phase [1,2]. The clinical manifestation of DKA encompasses symptoms such as dehydration, tachypnea, nausea, vomiting, abdominal pain, altered respirations, and a gradual decline in consciousness [3].

The genesis of DKA lies in insulin deficiency, precipitating an upsurge in counterregulatory hormones (e.g., glucagon, cortisol, and catecholamines) circulating systemically [4]. The synergy of insufficient insulin and heightened catabolic hormones leads to amplified glucose production through glycogenolysis and gluconeogenesis. Simultaneously, there is diminished glucose utilization by peripheral tissues and an escalation in lipolysis and ketogenesis [1]. These metabolic processes may culminate in hyperglycemia, ketonuria, and metabolic acidosis. The resultant hyperglycemia induces osmotic diuresis, severe dehydration, and electrolyte loss [5]. Without timely correction through intravenous insulin and fluid/electrolyte therapy, this condition can progress to fatal metabolic acidosis and eventual demise.

The prevailing diagnostic criteria for DKA encompass a blood glucose level exceeding 250 milligrams/deciliter (mg/dL), a venous pH below 7.3, and/or a bicarbonate level lower than 18 millimoles per liter (mmol/L), coupled with the presence of moderate or large ketones in the urine (≥3+ ketones) [6]. While not obligatory for diagnostic purposes, the measurement of serum beta-hydroxybutyrate can be undertaken, and a concentration equal to or exceeding 3 (mmol/L) is indicative of DKA [1]. Our case highlights a non-classic presentation of DKA in a two-year-old female, which was recognized by her primary care pediatrician and treated promptly in the emergency department.

## Case presentation

Our patient, a two-year-old female, had an uncomplicated birth history through spontaneous vaginal delivery at 39 weeks to a Gravida two Para two* *(G2P2) female with gestational diabetes. She presented to Urgent Care with a two-day history of abdominal pain and fatigue. The patient was discharged from urgent care with a diagnosis of "unspecified abdominal pain," and the mother was advised to "continue to monitor the patient." Over the next four days, the patient's abdominal pain worsened, prompting her mother to bring her in for evaluation at her primary care physician's office. The mother reported no new symptoms but stated that the patient overall "had not been looking good." A urinalysis conducted in the office revealed urine glucose >500 mg/dL, ketones of 100 mg/dL, and a serum glucose level of 282. Upon learning of the lab results and further questioning, the mother disclosed that the patient had been frequently wetting the bed and waking up at night asking for water. Subsequently, the patient was promptly referred to the emergency department for further evaluation and treatment. On arrival, she was afebrile (with a temperature of 36.7 degrees Celsius, tachycardic (with a heart rate of 114 beats per minute), and tachypneic (with a respiratory rate of 42 breaths per minute). Pertinent laboratory results (Table 1) reveal an elevated white blood cell count (likely due to inflammation from acute illness), pseudohyponatremia, and partially compensated metabolic acidosis.

**Table 1:** Pertinent lab results pCO_2_ = partial pressure of carbon dioxide; pO_2_ = partial pressure of oxygen; K/mm^3^ = thousand cells per cubic millimeter; mg/dL = milligrams per deciliter; mmol/L = millimoles per liter; mmHg = millimeters of mercury

Test:	Value:	Reference Range:
White Blood Cell Count	14.1 K/mm^3^	4.5-11.0 K/mm^3^
Glucose (Serum)	533 mg/dL	80-100 mg/dL
Sodium	129 mmol/L	135-145 mmol/L
Corrected Sodium	137 mmol/L	135-145 mmol/L
Potassium	4.3 mmol/L	3.5-5.0 mmol/L
Chloride	100 mmol/L	95-105 mmol/L
Magnesium	2.3 mg/dL	1.5-2.0 mg/dL
Calcium	9.6 mg/dL	8.4-10.2 mg/dL
Phosphorus	3.2 mg/dL	3.0-4.5 mg/dL
pH, Venous Blood Gas	7.086	7.32-7.43
pCO2, Venous Blood Gas	16.7 mmHg	40-60 mmHg
pO2, Venous Blood Gas	45.7 mmHg	30-55 mmHg on room air
Bicarbonate, Venous Blood Gas	7.7 mmol/L	22-27 mmol/L
Blood Urea Nitrogen (BUN)	15 mg/dL	7-18 mg/dL
Creatinine	0.30 mg/dL	0.6-1.2 mg/dL

Labs

In the emergency department, the patient was diagnosed with T1DM with severe DKA without coma. Subsequently, the patient was treated with intravenous fluids, insulin, and electrolytes. Following this intervention, the acidosis was corrected, blood glucose levels were lowered, and the patient was stabilized before being admitted to the pediatric intensive care unit (PICU).

At discharge, the patient was prescribed six units of injectable Lantus to be administered nightly, in addition to Humalog to be taken at meal times based on a carbohydrate count ratio of 1:15. Detailed instructions were provided for the patient to monitor blood sugar levels before each meal through fingerstick readings. Additionally, the patient's mother received education on accurate carbohydrate counting and underwent training on the appropriate use of syringes for insulin injections.

For long-term management, the patient was referred to endocrinology for comprehensive assessment and treatment. She has been under continuous monitoring since being diagnosed with T1DM at the age of two, up to her current age of eight.

At the age of three, the patient exhibited a hemoglobin A1C of 7.2, with an average blood glucose level of approximately 200. The patient consistently adhered to prescribed dosages, refrained from adjusting insulin doses between appointments, never utilized glucagon, and consistently monitored blood glucose and ketones using the Dexcom G6 continuous glucose monitor (San Diego, California, USA).

At the age of five, the patient experienced a new-onset seizure upon waking, prompting a referral to neurology by her pediatrician. Subsequent investigation revealed that the cause of the seizure-like activity was hypoglycemia, as confirmed by a normal electroencephalogram.

At the age of seven, the patient contracted COVID-19. Following this, she sought emergency care due to elevated ketones, a hemoglobin A1C of 10.9, and an average blood glucose level of 293.

Three months later, during a follow-up with the endocrinologist, the patient reported challenges in controlling blood glucose levels, which showed improvement with hyperglycemia corrections. The sole modification to her treatment involved adjusting her bolus insulin dose at meal times. Furthermore, the patient received counseling on proper injection site usage due to the development of new-onset lipodystrophy.

Currently, at eight years old, the patient has a hemoglobin A1C of 9.7%, average blood glucose of approximately 276, and persistent elevation of glucose above 200 throughout the day, dropping to the 80s during nocturnal sleep. During the latest endocrine visit, minor adjustments were made to her bolus doses at meal times. She will continue regular follow-ups with endocrinology to achieve tighter glycemic control.

## Discussion

Delayed diagnosis of T1DM increases the risk of developing the acute complication, DKA. This condition is emergent, life-threatening, and requires intensive treatment. Additionally, DKA is often the first symptom of previously undiagnosed diabetes in children. Prompt diagnosis and appropriate management are essential to prevent long-term sequelae and fatality [7].

The standard diagnostic criteria for DKA include a blood glucose level surpassing 250 milligrams/deciliter (mg/dL), a venous pH below 7.3, and/or a bicarbonate level below 18 millimoles per liter (mmol/L), alongside ketonemia or ketonuria. DKA can be further classified as mild, moderate, or severe. Mild DKA is characterized by a pH ranging from 7.25 to 7.3 and a serum bicarbonate level between 15 and 18 mEq/L. Moderate DKA is marked by a pH between 7.0 and 7.24 and a serum bicarbonate level of 10 to less than 15 mEq/L. Lastly, severe DKA is classified by a pH below 7.0 and bicarbonate levels below 10 mEq/L [8].

Strict glycemic control with intensive insulin therapy is necessary for the long-term management of T1DM to prevent fatal hyperglycemic complications and to delay the progression of vascular disease. The two mainstays of therapy include the use of rapid (bolus) and long-acting (basal) insulin delivered subcutaneously via injections or through subcutaneous insulin infusion via pumps. Individuals who self-administer insulin must exercise caution, as improper bolusing can precipitate the development of DKA. Therefore, patients must monitor their blood glucose daily for effective management [9].

As displayed in the case, patient management was excellent based on blood glucose monitoring, medication compliance, and physician/parent involvement. However, persistent hyperglycemia and a significantly elevated hemoglobin A1C, consistently near 10%, exhibit poor patient control. It is important to note that the patient experienced two major complications during the long-term management of her condition; the first was a hypoglycemic seizure and the second was acute illness (contraction of COVID-19).

Currently, the treatment and management of diabetes are suboptimal due to the risk of iatrogenic hypoglycemic seizures that can occur due to hyperglycemic corrections with short-acting insulin [10]. Commonly, hypoglycemic seizures may occur when diabetic patients are asleep due to the inability to sense the warning signs of low blood sugar, which include headache, pallor, sweating, shakiness, nausea, tachycardia, etc. [11]. Hypoglycemia depletes the brain of its primary fuel, which may result in functional brain failure. Often, this can be corrected by increasing plasma glucose; however, in severe cases, it may result in cerebral edema, neuronal death, coma, and fatality [12].

As seen with this patient's case, acute illness (contraction of COVID-19) appeared to play a role in the exacerbation of her T1DM via a rise in ketones, an A1C over 10%, and an inability to manage blood glucose levels. A *T1D Exchange *study conducted in 2021* *coordinated with 46 disease centers in which pediatric COVID-19 cases were collected from 266 patients with previously established T1DM. DKA proved to be the most common acute complication in 72% of hospitalized patients [13]. While the mechanism is not clearly understood, interleukin-six levels are elevated in both COVID-19 cases and in patients with DKA, which proposes a potential influence of the inflammatory cytokines that are released during viral illness in the precipitation of DKA [14].

## Conclusions

As exhibited in our case, prompt diagnosis and effective management are crucial for patients who present with new-onset, T1DM with ketoacidosis. Physicians need to consider DKA as a potential diagnosis for any pediatric patient presenting with abdominal pain. Additionally, this case emphasizes the importance of providing point-of-care glucose tests in all urgent care facilities. In the acute setting, our patient was appropriately treated with intensive insulin therapy and corrections for fluid and electrolyte imbalances. Her continued long-term care will necessitate consistent follow-up, medication compliance, and a dedicated commitment to achieving stringent glycemic control.
